# Remarkable therapeutic effects of methylphenidate in periodic limb movement disorder comorbid with excessive daytime sleepiness: a case report

**DOI:** 10.3389/fnins.2025.1659120

**Published:** 2025-10-09

**Authors:** Jiafeng Ren, Xinyan Zhang, Junying Zhou, Liu Liu

**Affiliations:** ^1^Sleep Medicine Center, West China Hospital, Sichuan University, Chengdu, China; ^2^Department of Neurology, West China Hospital, Sichuan University, Chengdu, China; ^3^Department of Anesthesiology, Sichuan Provincial People’s Hospital, School of Medicine, University of Electronic Science and Technology of China, Chengdu, China

**Keywords:** periodic limb movement disorder, periodic leg movement index, excessive daytime sleepiness, methylphenidate, case report

## Abstract

**Background:**

Periodic limb movement disorder (PLMD) is a sleep-related movement disorder, which may be accompanied by a complaint of excessive daytime sleepiness (EDS). With this study, we reported a clinical therapy process up to 2.5 years follow-up, exploring the therapeutic effect of a series of medicines in PLMD comorbid EDS.

**Case presentation:**

A 27-year-old woman suffered from EDS for more than 10 years and was then diagnosed with PLMD. Initially, we used levodopa 250 mg per night for 12 weeks, then piribedil 50 mg per night was arranged as the second drug for 12 weeks, and the third drug, pramipexole, was prescribed at a dosage of 0.25 mg per night for 12 weeks, but the EDS was persistent despite the periodic leg movement index (PLMI) returning to normal. Finally, methylphenidate was prescribed at a dosage of 18 mg per day after 4 weeks of washout. Then, the indicators of EDS and PLMD all returned to normal after 6 weeks. A 2.5-year follow-up was scheduled for taking methylphenidate; the symptoms of EDS were steadily well controlled, and PLMI was still kept in the normal range.

**Conclusion:**

This finding suggests that methylphenidate is an optimal therapeutic approach for the management of PLMD comorbid EDS.

## Introduction

Periodic limb movement disorder (PLMD) is a sleep-related movement disorder characterized by clinical sleep disturbance or fatigue attributed to an increased number of periodic limb movements of sleep (PLMS), in the absence of alternative causes of the sleep disorder (American Academy of Sleep Medicine, 2014; Lee et al., 2015). The diagnostic criteria for PLMD are periodic leg movement index (PLMI) > 5/h in children or >15/h in adults, in conjunction with nighttime sleep disturbances, excessive daytime sleepiness (EDS), mood disorders, or impairment in other important areas of functioning (American Academy of Sleep Medicine, 2014). Previous epidemiological studies reported that the prevalence of PLMD is 3.9% in general adults (Lee et al., 2015; Hornyak et al., 2006) with a slight predominance of females (Ohayon and Roth, 2002). Dopaminergic agents are primarily administered to treat PLMD (Aurora et al., 2012; Vignatelli et al., 2006); unfortunately, it could lead to a high risk of side effects (e.g., sleepiness) or augmentation develops after long-term usage (Aurora et al., 2012; Hwang et al., 2019). Currently, most interventional studies of dopaminergic agents on PLMD with EDS mainly report the improvement of PLMS, but lack information about the effects on EDS (Hornyak et al., 2006). Consequently, therapeutic measures are under challenge in patients with PLMD comorbid with EDS. Methylphenidate, a commonly used drug for attention-deficit hyperactivity disorder (ADHD), is also well known for improving the symptoms of EDS (Faraone et al., 2015; Banerjee et al., 2004). In the current study, we conducted a series of drug therapies in a patient with severe EDS caused by PLMD, under a designed clinical therapy process lasting 2.5 years of follow-up, suggesting the remarkable therapeutic effect of methylphenidate.

## Case presentation

A 27-year-old woman suffered from significant EDS for more than 10 years, and the symptoms got worse over the months. In detail, the patient complained of EDS in the morning, even though after a sufficient nighttime sleep of 7 to 8 h, the symptom did not improve after taking naps. Moreover, the EDS symptoms obviously impaired her job performance and further compromised her global daily function. She worked as an office clerk, a role that required frequent participation in meetings and focused document processing. Before treatment, EDS caused recurrent drowsiness during meetings and markedly reduced work efficiency. Notably, she had no complaints of snoring, cataplexy, sleep paralysis, hypnagogic hallucinations, or disrupted nocturnal sleep, including restless legs syndrome. She had a normal circadian rhythm and reasonable nighttime sleep duration (< 9 h) even under the unrestricted sleep schedule. Regarding her lifestyle, she engaged in mild weekly exercise (three 30-min sessions of brisk walking) and avoided excessive caffeine or alcohol intake. She also had no history of physical diseases, mood or psychotic disorders, medication use, or substance abuse. She lived with her partner and described stable interpersonal relationships in the absence of major work-related stress. Additionally, no family history of genetic or other significant disorders was reported. Due to the worsening of EDS symptoms, she attended the sleep clinic at the West China Hospital of Sichuan University. No prior targeted interventions (pharmacological or non-pharmacological) for EDS were attempted before this visit. Routine physical examinations found no obvious neurologic or psychiatric abnormalities, and her body mass index (BMI) was 18.7 kg/m^2^. The brain MRI findings were unremarkable. The scores on the Hamilton depression scale-17 items and the Insomnia Severity Index were within normal limits. However, she scored 16 on the Epworth Sleep Scale (ESS), which indicated severe EDS. To evaluate the objective nocturnal sleep pattern and daytime sleepiness, overnight polysomnography (PSG) followed by a multiple sleep latency test (MSLT) was performed at baseline. The results of PSG revealed a normal sleep pattern, in which the total sleep time (TST) was 441.5 min and sleep efficiency (SE) was 91.8%, except for the abnormal PLMI being 60.1 events/h. The MSLT result showed that the mean sleep latency (MSL) was 7.4 min, which confirmed the objective of EDS in line with the subjective finding from ESS. Notably, no sleep-onset rapid eye movement period (SOREMp) was observed in MLST. Therefore, based on the coexistence of PLMS and EDS, we made the diagnosis of PLMD for this patient according to the standard criteria in the International Classification of Sleep Disorders, third edition (ICSD-3rd) (American Academy of Sleep Medicine, 2014).

The patient was first prescribed levodopa at a dosage of 250 mg per night. After 2 weeks, a significant improvement in subjective sleepiness was reported with a score of 9 in ESS; however, the MSL was still 6.4 min in MSLT. Furthermore, at this time point (second week), a sharp drop in PLMI was from 60.1 to 15.9 events/h in PSG. Then the patient continuously took levodopa for the following 10 weeks. The reassessments (12th week) showed that PLMI was normal with 10.6 events/h, while MSL was consistently shortened to 2.7 min, indicating a deteriorated objective EDS. As well, the patient was unsatisfied with the persistent daytime sleepiness during the treatment of levodopa, particularly dozing episodes during work meetings (ESS Item 3: sitting inactive in a public place; still rated 3), despite a stable total ESS score of 9. Then, the second drug, Piribedil (a dopamine receptor agonist) was chosen to assess the therapeutic effect for this patient at 50 mg per night. In the 16th week, the results showed consistent EDS both in ESS (13 scores) and MSLT (MSL = 4.5 min). During the following 8 weeks, the continuous treatment of Piribedil did not alleviate the EDS symptom; even worse, the PLMI increased to 22.6 events/h. Another dopamine receptor agonist, pramipexole, was prescribed at a dosage of 0.25 mg per night in the 24th week. However, the subjective and objective EDS did not improve at the two follow-up timepoints (34th and 36th weeks) despite the PLMI returning to normal.

Although the dopaminergic agents showed optimal effects on ameliorating PLMS, the subjective and objective symptoms of EDS in this study persisted. Moreover, the patient experienced sleep attacks and more than two SOREMp were reported in MSLT after receiving dopamine agonist therapy. To address this concern, methylphenidate, a central nervous system stimulant, was evaluated for its effect on EDS secondary to PLMS. After a 4-week washout (40th week), the symptoms of EDS and PLMS were worsened (ESS = 14, PLMI = 51.6 events/h). Then, the methylphenidate was prescribed at a dosage of 18 mg per day, followed by short- and long-term assessments on PLMS and EDS. After 2 weeks of treatment with methylphenidate (42nd week), the subjective (ESS = 9) and objective (MSL = 10 min) EDS were significantly improved, and the PLMI was also decreased to 29.5 events/h. Notably, ESS Item 3 was reduced to 0, and the patient reported complete resolution of drowsiness during the work meetings. Afterward, we repeated the assessment 6 weeks of treatment later (46th week), and the indicators of EDS (ESS = 8, MSL = 10.8 min) and PLMS (PLMI = 11.0 events/h) returned to normal. Consequently, the patient was administered long-term methylphenidate (18 mg/day) and experienced an effective therapeutic response. After continuously taking methylphenidate for 2.5 years (167th week), a follow-up was performed to evaluate the symptoms of EDS and PLMS. The patient had no complaint of daytime sleepiness (ESS = 8) and returned to normal activities. The indicators in overnight PSG and MSLT were almost kept within the normal range (PLMI = 17.1 events/h, MSL = 11.2 min). Throughout the entire treatment course, she strictly adhered to the medication regimen with no missed doses and attended all scheduled assessments.

**Figure 1:**
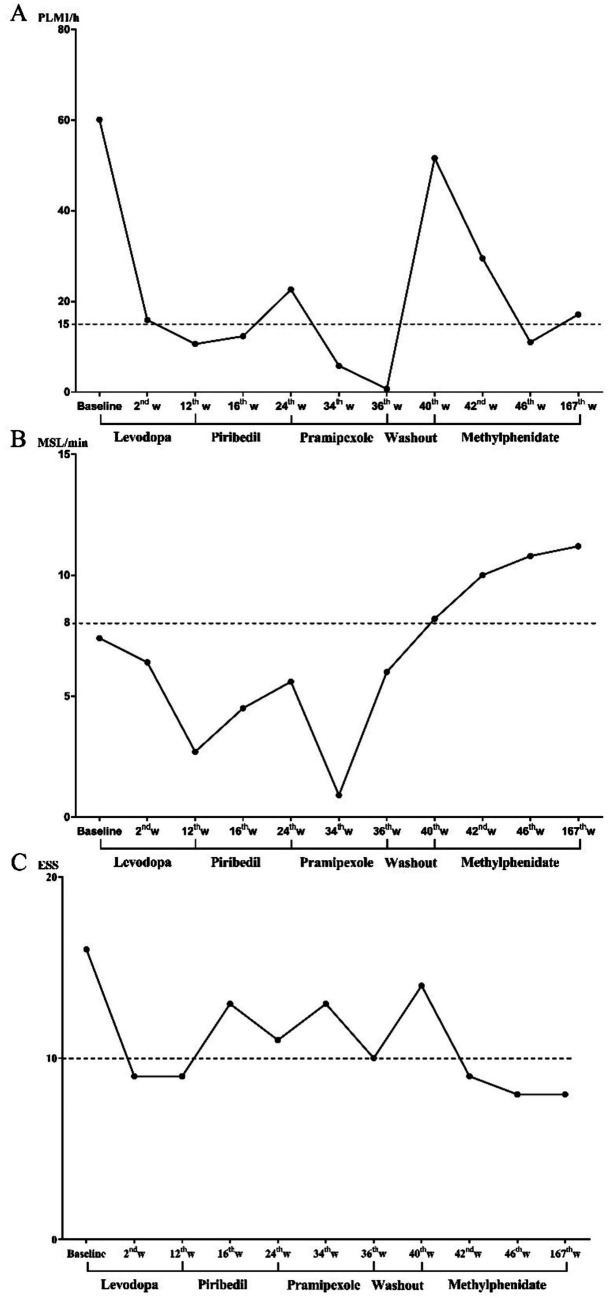
Changing process of subjective and objective EDS and PLMI during drug treatment. **(A)** The changing process of PLMI, **(B)** changing process of MSL, and **(C)** changing process of ESS. EDS, excessive daytime sleepiness; ESS, Epworth sleeping scale; MSL, mean sleep latency in multiple sleep latency test; PLMI, periodic limb movement index.

Additionally, no obvious side effects were reported in this patient under the treatment procedure, and the dosage of methylphenidate remained unchanged during the follow-up. Although we observed a decrease in slow wave sleep (N3) after administering methylphenidate, the other parameters of sleep architecture, including TST, SE, and wake after sleep onset, were basically normal, and the patient kept being satisfied with her sleep quality. All the results of sleep assessments are presented in Figure 1 and Table 1.

**Table 1:** Timeline of medication use, subjective, and objective sleep parameters.

Timeline	Baseline	2nd week	12th week	16th week	24th week	34th week	36th week	40th week	42nd week	46th week	167th week
Drugs	-	L-DOPA	L-DOPA	Piribedil	Piribedil	Pramipexole	Pramipexole	Washout	MPH	MPH	MPH
Treatment duration	-	2 w	12 w	4 w	12 w	10 w	12 w	-	2 w	6 w	127 w
Daily drug dose (mg)	-	250	250	50	50	0.25	0.25		18	18	18
PSG											
TST (min)	441.5	485.5	504.5	440	382.5	504.5	426.5	418.5	469.5	483.5	454.9
SL (min)	22	12.5	10	17.5	14.5	5	13.5	14.5	10	3.5	18
WASO (min)	14	19	8.5	15.5	17	19.5	15	7.5	12.5	28	14.5
SE (%)	91.8	93.7	96.5	93	92.4	95.4	93.9	95.3	95.4	93.9	93.5
N1 (%)	6.3	11.2	10.5	13	17.4	11.6	18.3	10.6	8.8	12.7	15.3
N 2 (%)	47.7	52.5	56.2	62.3	57.1	59.8	60.0	52.7	70.1	60.7	63.3
N3 (%)	18.5	20.8	16	12.4	6.4	15.1	7.5	18.2	2.6	9.8	2.1
R (%)	27.5	15.4	17.3	12.4	19.1	13.6	14.2	18.5	18.5	16.8	19.3
AHI /h	3.4	0.7	0.5	0.3	0.6	1.7	0.7	2.7	1.7	4.7	1.8
AI /h	19	14.5	25.8	11.5	11	16.7	10.7	16.2	14.2	13.8	10.3
PLMI /h	60.1	15.9	10.6	12.3	22.6	5.8	0.7	51.6	29.5	11.0	17.1
ESS	16	9	9	13	11	13	10	14	9	8	8
MSLT											
MSL (min)	7.4	6.4	2.7	4.5	5.6	0.9	6.0	8.2	10.0	10.8	11.2
SOREMp (n)	0	1	1	1	2	3	2	1	1	0	1

AHI, apnea-hypopnea index; AI, arousal index; ESS, Epworth sleeping scale; L-DOPA, levodopa; MPH, methylphenidate; MSL, mean sleep latency; MSLT, multiple sleep latency test; N, non-rapid eye movement sleep; PLMI, periodic limb movement index; R, rapid eye movement sleep; SE, sleep efficiency; SL, sleep latency; SOREMp, sleep-onset rapid eye movement period; TST, total sleep time; WASO, wake after sleep onset.

## Discussion

This study illustrated a therapeutic process for a patient with PLMD comorbid with EDS. The dopaminergic agents, as the first-line therapy approach for PLMD, are well-documented in reducing PLMI; conversely, they exacerbated EDS, although the PLMS improved in this study. To address the challenge, methylphenidate was administered with close supervision and long-term follow-up, and then the patient exhibited remarkable improvements in both PLMS and EDS. Additionally, no obvious adverse effects were reported, and the dosage of methylphenidate was stable even after a persistent treatment. Consequently, methylphenidate could be considered a candidate treatment option for patients with PLMD comorbid with EDS.

The treatment recommendations for PLMD are primarily levodopa and dopamine agonists (Aurora et al., 2012; Vignatelli et al., 2006; Hening et al., 2004), which alleviate PLMI by enhancing extracellular dopamine or activating D2/D3 dopamine receptors in the striatum (Manconi et al., 2011; Manconi et al., 2021). However, it is noteworthy that dopaminergic drugs, especially dopamine agonists, were reported with side effects such as daytime somnolence and sleep attacks affecting up to 50% of patients (Knie et al., 2011; Gieteling et al., 2012). Our findings support that dopaminergic drugs could exacerbate both subjective and objective EDS in line with the previous results. In this case, first-line dopaminergic agents (e.g., levodopa, piribedil, and pramipexole) effectively reduced PLMI (e.g., levodopa decreased PLMI from 60.1 to 10.6 events/h), yet consistently worsened EDS (e.g., levodopa shortened MSL from 7.4 to 2.7 min). Some agents also induced SOREMPs during MSLT. Given that the core complaint of EDS remained unresolved in this study, a more targeted therapy was conducted to alleviate EDS caused by PLMD. Consequently, we determined to use methylphenidate, a central nervous system stimulant commonly used in the treatment of EDS in narcolepsy (Banerjee et al., 2004). This drug is also used in ADHD (Faraone et al., 2015), which could elevate synaptic concentrations of dopamine and noradrenaline by inhibiting the reuptake of presynaptic dopamine transporter (DAT) and noradrenaline transporter, particularly in the striatum and frontal cortex (Salatino-Oliveira et al., 2011; Madras et al., 2005; Moreau et al., 2012). At a dose of 18 mg/day, it not only normalized EDS (ESS decreased from 14 to 8; MSL improved from 7.4 to 11.2 min) but also maintained PLMI within normal limits (11.0–17.1 events/h) over 2.5 years. In the current study, we speculated that methylphenidate improved the symptoms of EDS and PLMS through two possible mechanisms. First, the enhancement of extracellular noradrenaline concentration in the brain might exhibit positive effects on reducing sleepiness (Madras et al., 2005). This potential mechanism aligns with the patient’s improvement in both subjective and objective EDS after methylphenidate administration. Second, previous studies have indicated that therapeutic doses of methylphenidate can block over 50% of dopamine transporters in the brain striatum (Volkow et al., 1998), which may increase extracellular dopamine levels in DAT-rich brain regions. This may be the reason for the improvement in PLMS after methylphenidate treatment. Our findings indicate that methylphenidate could be an optimal therapeutic intervention for PLMD comorbid with EDS based on its potential efficacy in this study.

Additionally, we were initially concerned about the potential adverse effects of methylphenidate on nocturnal sleep. In practice, the patient did not complain about sleep disturbances and maintained a relatively normal sleep architecture. Notably, methylphenidate showed no apparent side effects even after long-term treatment. In addition, it is noteworthy that the patient always maintained satisfactory nighttime sleep quality throughout the disease course, and the likelihood of EDS attributed to other sleep disorders had been ruled out. These evidences not only validate our diagnosis but also reveals the efficacy of methylphenidate in PLMS apart from EDS. Notably, methylphenidate was well-tolerated in this patient over 2.5 years, with no observed adverse effects such as dependence, anxiety, or hypertension. However, certain risks must be considered. For instance, it may carry an abuse potential linked to activating the dopamine reward pathway, along with psychiatric effects (e.g., insomnia) and cardiovascular changes (e.g., elevated blood pressure). Thus, larger long-term studies are still necessary to fully establish the safety profile of methylphenidate for PLMD comorbid with EDS.

## Conclusion

This study is the first to propose that methylphenidate has a notable impact on patients with PLMD comorbid with EDS. We have established the short-term and long-term efficacy of methylphenidate, which suggests its unique therapeutic values for EDS attributed to PLMS. However, it is crucial to acknowledge that in-depth research is warranted to establish the efficacy of methylphenidate in PLMD and explore the underlying mechanisms. Longitudinal and randomized controlled trials should be conducted to verify our results and strengthen the current understanding of methylphenidate in the context of PLMD treatment.

## Data Availability

The raw data supporting the conclusions of this article will be made available by the authors, without undue reservation.
